# Construction and Evaluation of a HCoV-OC43 S2 Subunit Vaccine Fused with Nasal Immuno-Inducible Sequence Against Coronavirus Infection

**DOI:** 10.3390/cimb47050355

**Published:** 2025-05-13

**Authors:** Hiraku Sasaki, Hiroki Ishikawa, Ayako Shigenaga, Yoshio Suzuki, Masayuki Iyoda

**Affiliations:** 1 Graduate School of Health and Sports Science, Juntendo University, Inzai 2701695, Chiba, Japan; yssuzuki@juntendo.ac.jp; 2 Department of Microbiology and Immunology, Showa Medical University Graduate School of Medicine, Shinagawa-ku, Tokyo 1428555, Japan; iyoda@med.showa-u.ac.jp; 3 Institute of Health and Sports Science & Medicine, Graduate School of Health and Sports Science, Juntendo University, Inzai 2701695, Chiba, Japan; ayamatsu@juntendo.ac.jp; 4 Division of Nephrology, Department of Medicine, Showa Medical University Graduate School of Medicine, Shinagawa-ku, Tokyo 1428666, Japan

**Keywords:** HCoV-OC43, SARS-CoV-2, S2 subunit, intranasal vaccine, neutralizing antibody

## Abstract

A partial sequence of an human coronavirus (HCoV)-OC43 S2 subunit that cross-reacts with the S2 subunit of severe acute respiratory syndrome coronavirus 2 (SARS-CoV-2) was fused with a nasal immuno-inducible sequence (NAIS), and the resulting complex was used for intranasal immunization of rabbits. Crude serum from rabbits immunized with three doses showed an IgG titer > 1000 against the S2 subunits of HCoV-OC43 and SARS-CoV-2 and inhibited OC43 viral replication as a neutralizing antibody in vitro.

## 1. Introduction

Intranasal vaccination is an effective method for preventing invasion and colonization of pathogens by eliciting secretory IgA (sIgA). Because live-attenuated vaccines are generally used as antigens for intranasal vaccination, immunization restrictions are imposed for safety reasons. However, the use of inactivated vaccines as antigens can improve vaccine safety and the range of vaccination targets can be expanded [1]. Since adjuvants or immunomodulators inoculated along with antigens play important roles during intranasal vaccination of an inactivated candidate, we developed an immunomodulatory collagen-binding protein MP3, derived from bacteria, with low cytotoxicity and extracellular matrix adhesion properties [2,3]. A further developed polypeptide sequence termed as a nasal immuno-inducible sequence (NAIS) elicited antigen-specific serum IgG and sIgA response after three or more intranasal vaccinations in a mouse study; therefore, the NAIS fused with antigens could be applied as an immunomodulator [3].

The receptor-binding domain (RBD) of the S protein is mainly targeted to develop vaccines for severe acute respiratory syndrome coronavirus 2 (SARS-CoV-2) to prevent infection and pathophysiology. Elicitation of antibody (Ab) response targeting the RBD prevents viral attachment to their receptor, angiotensin-converting enzyme 2 (ACE2) [4]; therefore, the RBD is an effective antigenic target to inactivate invading viruses. Considering the situation of changing variants, conducting experiments with different antigenic and vaccine designs is necessary. For human coronaviruses (HCoVs), neutralizing Abs (nAbs) against the S protein inhibits viral attachment by binding to the S1 subunit and virus–cell membrane fusion by binding to the S2 subunit, therefore, preventing the entry and transmission of HCoVs [5]. The S2 subunit is a potential target of nAbs that interferes with the structural rearrangement of the S protein and virus–host membrane fusion [6]. Therefore, nAbs against the S2 subunit may prevent viral membrane fusion to further inhibit viral replication. Furthermore, the C-terminus of the S2 subunit is relatively conserved between SARS-CoV-2 and seasonal HCoVs.

In this study, we fused the S2 subunit of HCoV with modified MP3 termed as the nasal immuno-inducible sequence (NAIS) and examined the effect of crude sera obtained by intranasal immunization of this complex on viral replication in vitro and studied its cross-reactivity with the S2 subunit of SARS-CoV-2.

## 2. Materials and Methods

### 2.1. Vaccine Design for Intranasal Immunization

Prior to designing NAIS and vaccine antigens, potential major histocompatibility complex (MHC)-I and -II binding site predictions were generated using the Immune Epitope Database (IEDB) [7]. For NAIS, potential antigenic sites were removed from MP3 while still preserving the collagen-binding ability as a following section [3]; for the S2 subunit antigen, the sites with concentrated epitope predictions were targeted and fused to the C-terminus of NAIS using L-arabinose-inducible pBAD vector [2]. In brief, the *E. coli* strain BL21-AI [2] harboring the expression vector was incubated at 37 °C for 2 h and then incubated at room temperature overnight after the addition of 0.02% L-arabinose (final concentration). Thereafter, the *E. coli* cells were collected by centrifugation (5000× *g*, 10 min) and sonicated. Six histidine tag fusion recombinant proteins were separated according to the manufacturer’s instructions using Dynabeads His-Tag Isolation & Pulldown (Thermo Fisher Scientific, Waltham, MA, USA). The separated proteins were then buffer-substituted in a solution of 50 mM Tris and 150 mM NaCl using a dialysis membrane and used for experiments.

### 2.2. Intranasal Immunization

Animal studies were conducted at Eurofins Genomics (Tokyo, Japan) in accordance with the recommendations of the Guide for the Care and Use of Laboratory Animals of the National Institutes of Health. The protocol was approved by the Institutional Animal Care and Use Committee (IACUC) of Eurofins Genomics under accession no. OA-2112023-00002. A 100 μL aliquot of 0.2 mg/mL NAIS-fused S2 antigen (NAIS-ag) was intranasally administered to single rabbit thrice at 1-week intervals for three weeks. At week 4, whole blood was collected, and the serum was separated and used as the test crude serum.

### 2.3. Enzyme-Linked Immunosorbent Assay (ELISA) and Western Blotting (WB)

ELISA was used to determine the collagen-binding ability and Ab titers. To determine the collagen-binding ability, a collagen type IV-coated 96-well plate (Corning, Bedford, MA, USA) was used for sandwich ELISA. Stepwise diluted OC43-S2 and NAIS-ag proteins were reacted on the plate for 2 h at room temperature, followed by five rigorous washes and detection using HRP-conjugated HisProbe (Thermo Fisher Scientific) and EzElisa TMB (Atto, Tokyo, Japan), according to the manufacturers’ instructions. For Ab titers, The HCoV-OC43 spike S2 (Sino biological, Beijing, China) and SARS-CoV-2 S2 (Raybiotech, Peachtree Corners, GA, USA) subunits were coated on 96-well plates overnight at 4 °C, followed by blocking with protein-free blocking buffer (Thermo Fisher Scientific), then reacted with diluted rabbit crude serum (2–1024) for 1 h at room temperature, followed by 5 washes, then reacted with HRP-conjugated anti-rabbit IgG (1: 5000, Promega, Madison, WI, USA) and anti-rabbit IgA pAb (1: 3000, Abcam, Cambridge, UK) and measured by EzElisa TMB. WB was used to determine the cross-reactivity between the vaccine antigen and immunized serum, using anti-OC43 spike glycoprotein polyclonal Ab (pAb, 1: 3000; Epigentek, Farmingdale, NY, USA) and anti-SARS-CoV-2 spike pAb (1: 300, Genetex, Alton Pkwy Irvine, CA, USA) as the primary antibodies. HRP-conjugated anti-rabbit IgG (1: 5000, Promega) and anti-rabbit IgA pAb (1: 3000, Abcam) were used as the secondary antibodies. When rabbit crude serum was used for WB, it was diluted 1:5000. The HCoV-OC43 spike S2 (Sino biological, Beijing, China) and SARS-CoV-2 S2 (Raybiotech, Peachtree Corners, GA, USA) subunits were used for comparative analyses. Although the molecular weight of the S2 subunit of both HCoV-OC43 and SARS-CoV-2 is predicted to be approximately 60 kDa, upon expression in eukaryotic cells, it may contain a membrane fusion component and show a molecular weight > 60 kDa. HCoV-OC43 S2 subunit was confirmed by LC-ESI-MS/MS analysis using a Thermo Fisher Scientific LXQ mass spectrometer with nano-liquid chromatography (AMR Inc., Tokyo, Japan)

### 2.4. Neutralization Assay of HCoV–OC43 Virus

HCT-8 cells were cultured at 2 × 10^5^ cells/well (200 µL media) density in Dulbecco’s Modified Eagle Medium (DMEM) supplemented with 10% fetal bovine serum in 96-well flat plate. OC43 virus at 1 × 10^4^ pfu/100 μL was mixed with 100 μL of unimmunized or immunized serum for 1 h at 37 °C. HCT-8 cells were then washed with DMEM and incubated with 100 μL of the virus mixture for 1 h at 37 °C. Cells were then washed thrice with DMEM and cultured for three weeks at 33 °C. Cytopathic effects and viral titers in the supernatants were estimated by the methods of Vijgen et al. [8] using a LightCycler 480 system (Roche diagnostics, Basel, Switzerland) in 5 replicates, following extraction of viral RNA using a MagMAX Viral RNA Isolation Kit (Thermo Fisher Scientific).

### 2.5. Statistical Analysis

Statistical analyses were performed using Microsoft Excel 8.0. Statistical comparisons were performed using an unpaired Student’s *t*-test. The values are presented as the mean ± standard deviation (SD). The *p* values < 0.05 were considered significant.

## 3. Results

**Figure 1:**
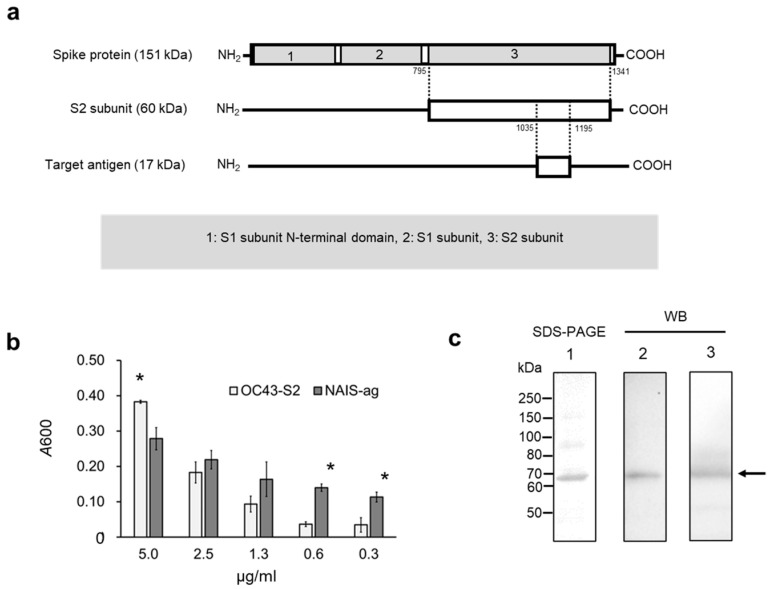
Characterization of the intranasal vaccine antigen derived from HCoV-OC43 S2 subunit. (**a**) 17 kDa fragment of the S2 subunit spanning 1035–1195 amino acid residues that contain many MHC-I and -II binding peptides predicted in the IEDB database. (**b**) Comparison of collagen type IV binding ability between the S2 subunit and NAIS-ag (NAIS fused with the 17 kDa fragment of the S2 subunit) at different concentrations. The S2 subunit showed significant binding only at 5 μg/mL concentration, whereas NAIS-ag showed significant binding at concentrations < 0.6 μg/mL (* *p* < 0.05). (**c**) Cross-reactivity of NAIS-ag against S proteins of both HCoV-OC43 and SARS-CoV-2. Lane 1 shows sodium dodecyl sulfate–polyacrylamide gel electrophoresis (SDS-PAGE) analysis of NAIS-ag, and lanes 2 and 3 show the results of WB using anti-HCoV-OC43 and anti-SARS-CoV-2 S protein pAb, respectively.

The 1035–1195 amino acid (aa) residues of the S2 subunit were fused to the NAIS to generate the vaccine NAIS-ag (Figure 1a). The MP3 and NAIS are characterized by their adhesiveness to collagen [3], and the NAIS-ag showed significantly higher adherence at low concentrations (≤0.6 μg/mL, *p* < 0.05) compared with the S2 subunit alone that showed adherence only at high concentrations (Figure 1b). The cross-reactivity of the NAIS-ag against the S proteins of HCoV-OC43 and SARS-CoV-2 was confirmed by Western blot analysis (Figure 1c).

**Figure 2:**
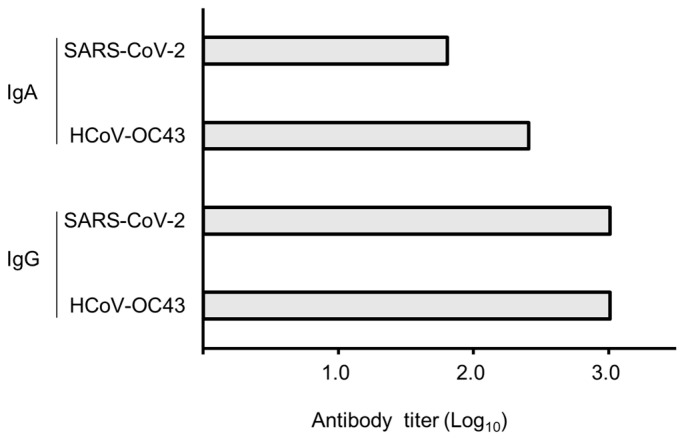
Antibody titers toward S2 subunits of both HCoV-OC43 and SARS-CoV-2. Results are shown in logarithm, and the maximum value was measured as 1024 (3.01 in log_10_).

Serum IgG titers obtained after three intranasal immunizations in rabbit against the S2 subunits of both HCoV-OC43 and SARS-CoV-2 were more than 1024 (Log_10_, 3.01), whereas those of serum IgA were 256 (2.41) and 64 (1.81) for HCoV-OC43 and SARS-CoV-2, respectively (Figure 2).

**Figure 3:**
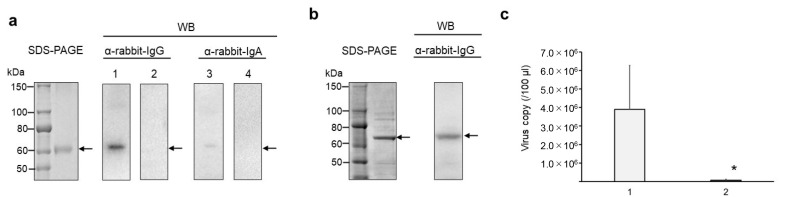
Characterization of crude serum obtained after three doses of intranasal immunization of NAIS-ag in a rabbit. (**a**) SDS-PAGE and WB analysis of HCoV-OC43 S2 subunit using crude serum as a primary antibody. Lanes 1 and 2 show immunized and unimmunized crude sera as primary Abs, respectively, detected with anti-rabbit IgG pAb. Lanes 3 and 4 show immunized and unimmunized crude sera as primary Abs, respectively, detected with anti-rabbit IgA pAb. (**b**) SDS-PAGE of SARS-CoV-2 S2 subunit and WB analysis using crude serum as a primary Ab and anti-rabbit IgG pAb as secondary antibody. The corresponding band was not confirmed using a secondary anti-rabbit IgA pAb. Arrows indicate the band position of the target protein. (**c**) Neutralization assay of HCoV-OC43 viral replication in HCT-8 cells using unimmunized (1) and immunized (2) crude sera. The viral titer test was performed in 5 replicates on a 96-well plate on a 200 µL scale. * *p* < 0.05.

WB analysis revealed the cross-reactivity of serum IgG, but not IgA, with the S2 subunits of HCoV-OC43 and SARS-CoV-2 (Figure 3a,b). Neutralization assays revealed that OC43 viral replication in HCT-8 cells was significantly inhibited by immunized serum (*p* < 0.05, Figure 3c). The intranasal immunization induced the production of nAbs that inhibited OC43 viral replication, further suggesting that it may be potentially effective against SARS-CoV-2.

## 4. Discussion

In this study, pAbs produced by rabbit were analyzed for coronavirus cross-activity. Rabbits have been reported to perform diverse genetic modifications with IgG [9]. In particular, changes in the Ig repertoire may be related to the greatest diversification compared to other animals [9]. The high IgG antibody titers observed with intranasal immunization may be a rabbit-specific immune response. Therefore, antibody titers may have been higher for IgG than for IgA. For collagen-binding capacity, it was observed that at low concentrations, the full-length S2 protein was higher than the NAIS-ag (Figure 1b). The full-length S2 used in this study contains a membrane-binding domain that is used during membrane fusion [10] and may have exhibited adhesion to collagen at high concentrations.

While using RBD as a vaccine target may be one effective means, the shortcomings caused by this vaccine design have also been hypothesized. The downregulation of Ab production against the viral RBD that binds to ACE2 may generate ACE2-binding complexes, therefore, resulting in adverse reactions after vaccination [11,12]. Furthermore, variants with several point mutations in the ORF1ab, N protein, and S1 subunit of S protein exist with previously reported variants [13], suggesting that the C-terminus of the S2 subunit is highly conserved among variants. Immunization with N-glycan shielded the S2 subunit from membrane fusion and may lead to less immune recognition and immunogenicity than that with the S1 subunit, and elicitation of nAbs following immunization with the S2 subunit is lower than that with the S1 subunit or RBD [14,15]. However, the C-terminus of the S2 subunit induces structural changes during membrane fusion, suggesting that it may be highly conserved and effective as a vaccine against SARS-CoV-2 variants and HCoVs.

Although bacterial toxins such as *Escherichia coli* heat-labile toxins and cholera toxins have been developed experimentally as immunomodulator adjuvants [16], the mechanism of these B subunits has been elucidated and it is understood that they are non-toxic and act as immunomodulatory proteins [17]. For the NAIS, parent MP3 is derived from a rodent opportunistic bacterial protein and is considered safe for humans due to delete the cytotoxic moiety [2]. Designing an intranasal vaccine containing the NAIS-ag to induce sIgA needs further clarification.

## Data Availability

The original contributions presented in the study are included in the article; further inquiries can be directed to the corresponding author.
